# Supplementary figures and images for: Chromatin remodeling of histone H3 variants by DDM1 underlies epigenetic inheritance of DNA methylation
Source: Cell. Author manuscript; Available in PMC 2023 Nov 28. (PMC10529913; doi:10.1016/j.cell.2023.08.001)

**A**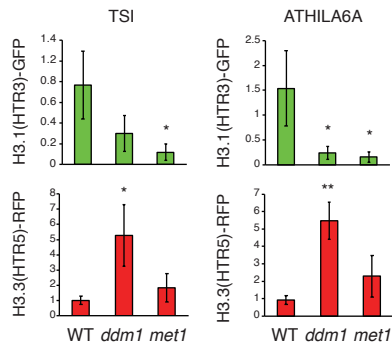**B**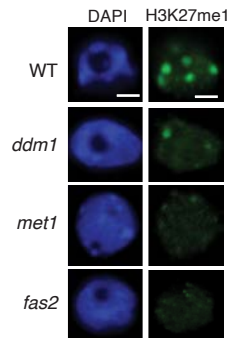**C**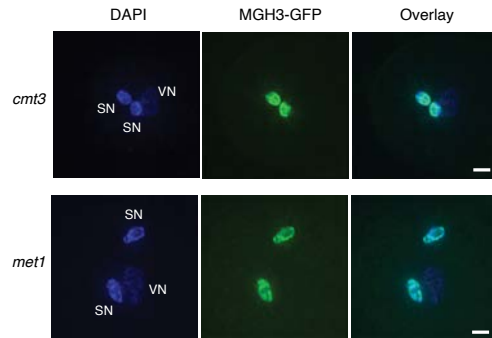**D**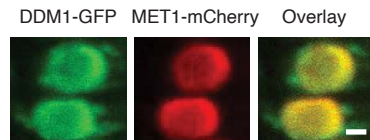**E**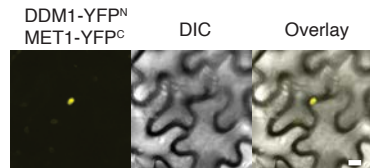**F**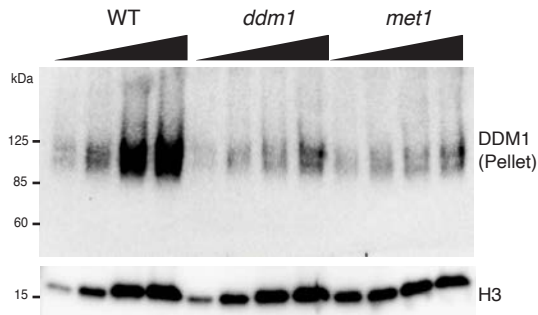**G**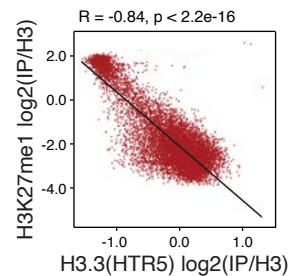

Supplement: 1 — Supplementary Figure S1. MET1 participates in histone remodeling by DDM1, related to Figure 1. (A) ChIP-qPCR amplification of TSI (ATHILA) and ATHILA6A repeats in wild-type (WT), ddm1, and met1 with 10-d-old seedling tissues. ChIP signals of H3.1(HTR3)-GFP and H3.3(HTR5)-RFP were normalized to H3. Error bars indicate standard deviations (biological replicates; n=3). P values of statistical difference with WT are shown above each mutant (one-way Anova adjusted with Tukey’s Honest Significant Difference method; * p-value<0.05, ** p-value<0.01). (B) Immunofluorescence of H3.1-associated histone modification H3K27me1 in 3-week-old leaves of WT, ddm1, met1, and fas2 (caf-1). DAPI was used for DNA staining. Scale bars indicate 2 μm. (C) Male Germline-specific Histone H3.3 MGH3-GFP localization in sperm nuclei of Arabidopsis pollen grains. DAPI staining was used to visualize vegetative (VN) and sperm nuclei (SN). Mislocalization to the nuclear periphery was observed in met1 mutants, but not in cmt3. Scale bars indicate 2 μm. (D) Co-localization of DDM1-GFP and MET1-mCherry in the nucleus. Scale bar indicates 2 μm. (E) Bimolecular fluorescence complementation using DDM1 fused with N-terminal YFP (YFPN) and MET1 with C-terminal YFP (YFPC). Scale bar indicates 5 μm. Complementation is defined by the yellow nucleus. (F) Western blot analysis of endogenous DDM1 from the chromatin/pellet (P) fraction of WT, ddm1, and met1 backgrounds. Anti-H3 was used as loading control. Serial dilutions (1:2) were made for each sample (gradient) indicating that both ddm1 and met1 mutants had between ½ and ¼ WT levels of chromatin-bound DDM1. (G) Genome-wide negative correlation between H3K27me1 (H3.1) and H3.3(HTR5)-RFP ChIP-seq in wild-type (Figure 1D). P and R values indicate statistical significance and Pearson correlation coefficients, respectively. [file NIHMS1922946-supplement-1.pdf]

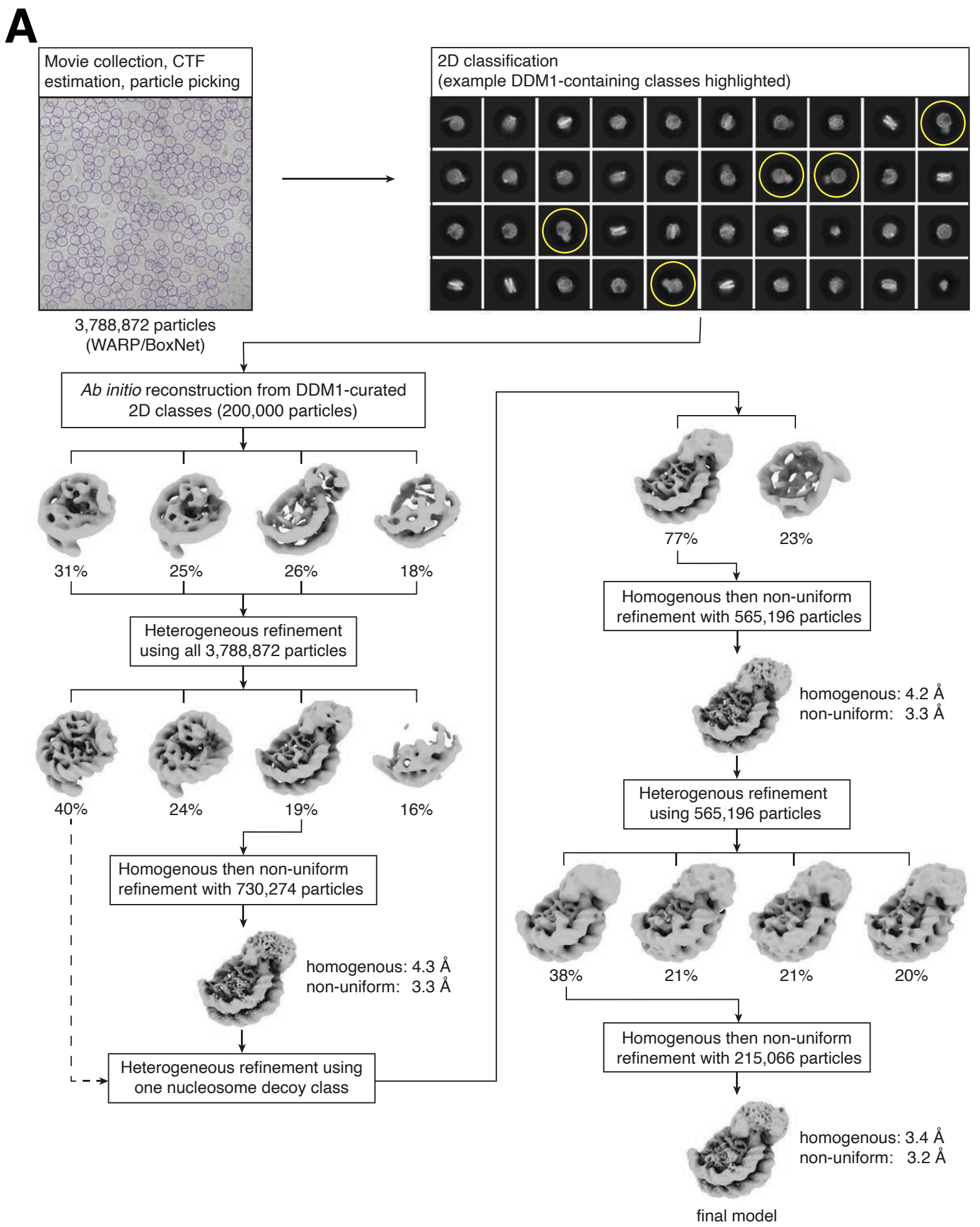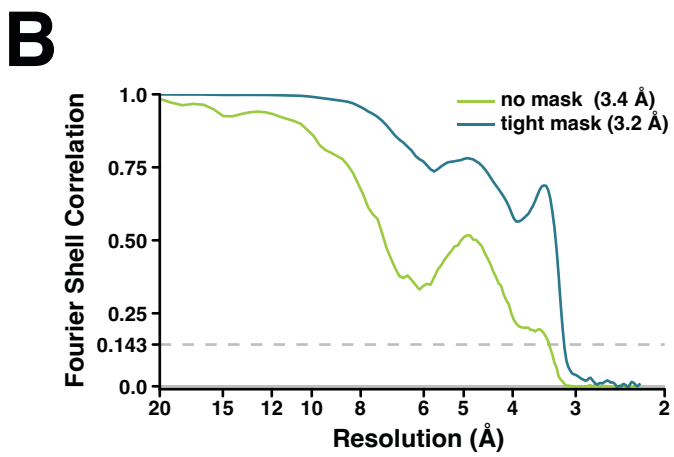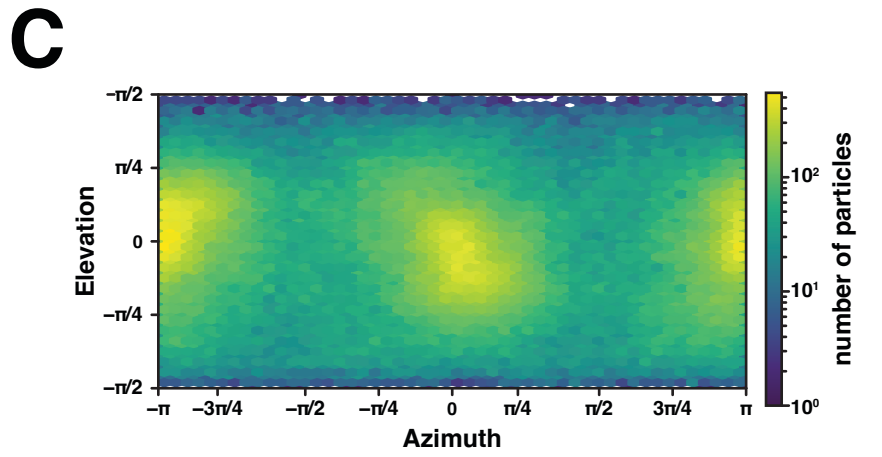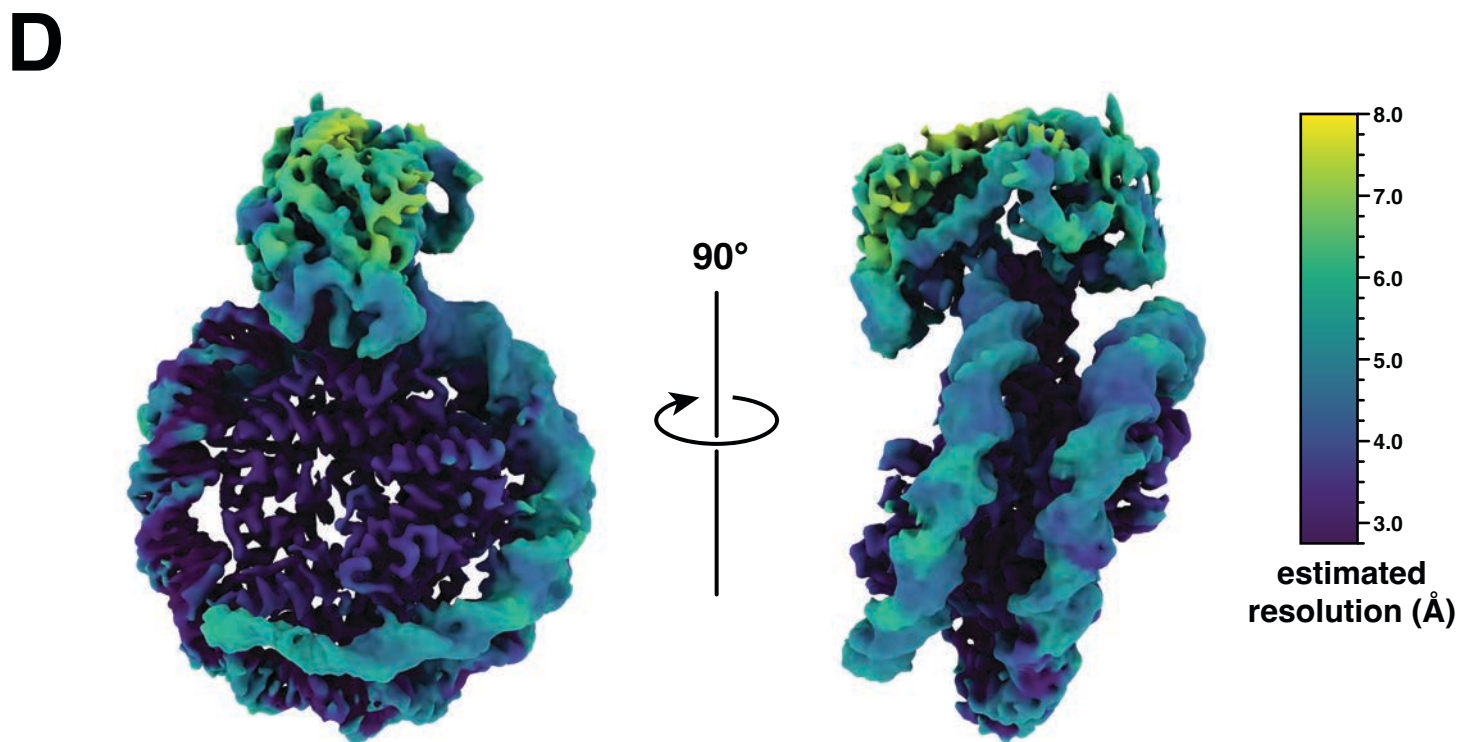

Supplement: 2 — Supplementary Figure S2. Cryo-EM data processing workflow and reconstruction metrics, related to Figure 3. (A) Following cryo-EM movie collection, motion correction, averaging, CTF estimation, and particle picking were performed using WARP96. Example particle picks are shown as purple circles (top left). Particles were then imported into cryoSPARC97 for 2D classification as well as 3D classification and refinement. Examples of DDM1-containing 2D classes, which were used to generate the ab initio models are highlighted with yellow circles (top right). Class distributions are indicated for each heterogenous refinement step. Reconstruction resolutions after homogenous and non-uniform refinement are indicated next to the corresponding models. (B) Fourier Shell Correlation (FSC) plots of the DDM1-nucleosome reconstruction using no mask (green) and a tight mask (blue). Resolution values at FSC 0.143 are indicated. (C) Angular distribution plot of reconstruction projections. The heat map indicates the number of particles per viewing angle. (D) The DDM1-nucleosome complex reconstruction, colored by estimated local resolution from cryoSPARC. [file NIHMS1922946-supplement-2.pdf]

**A**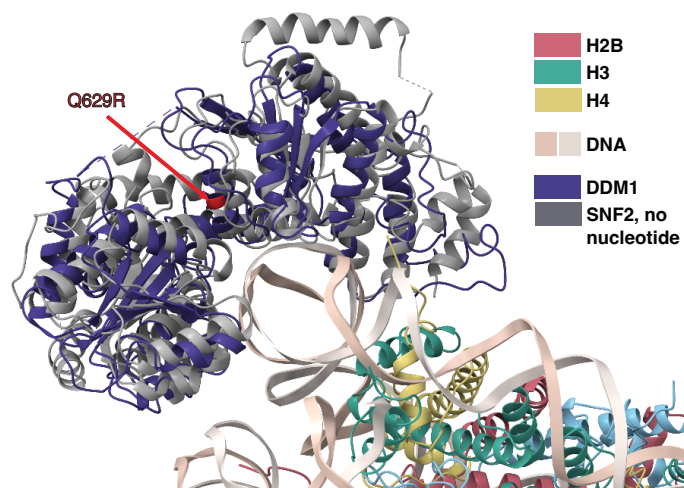**B**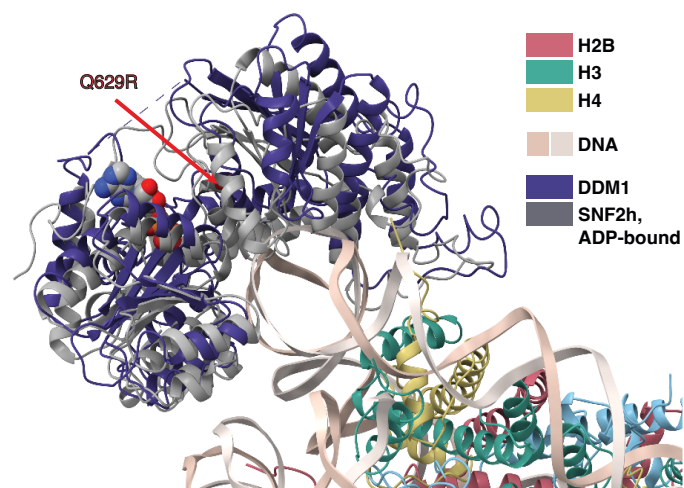**C**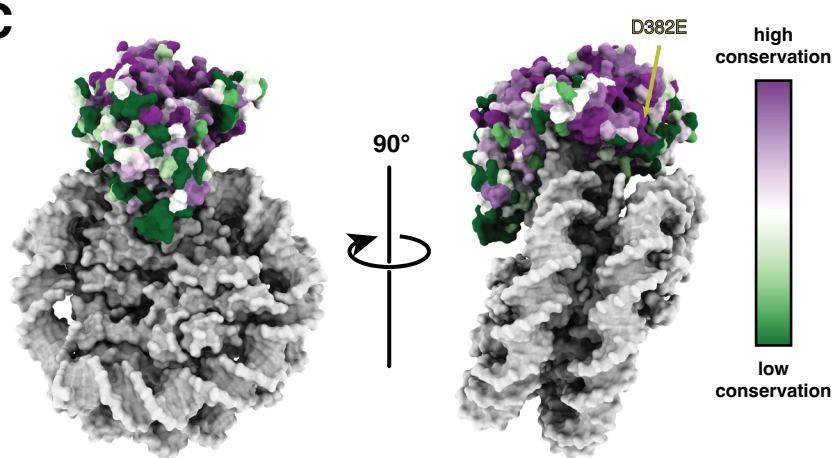**D**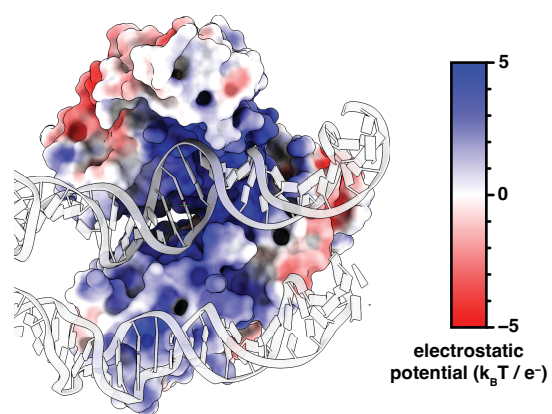**E**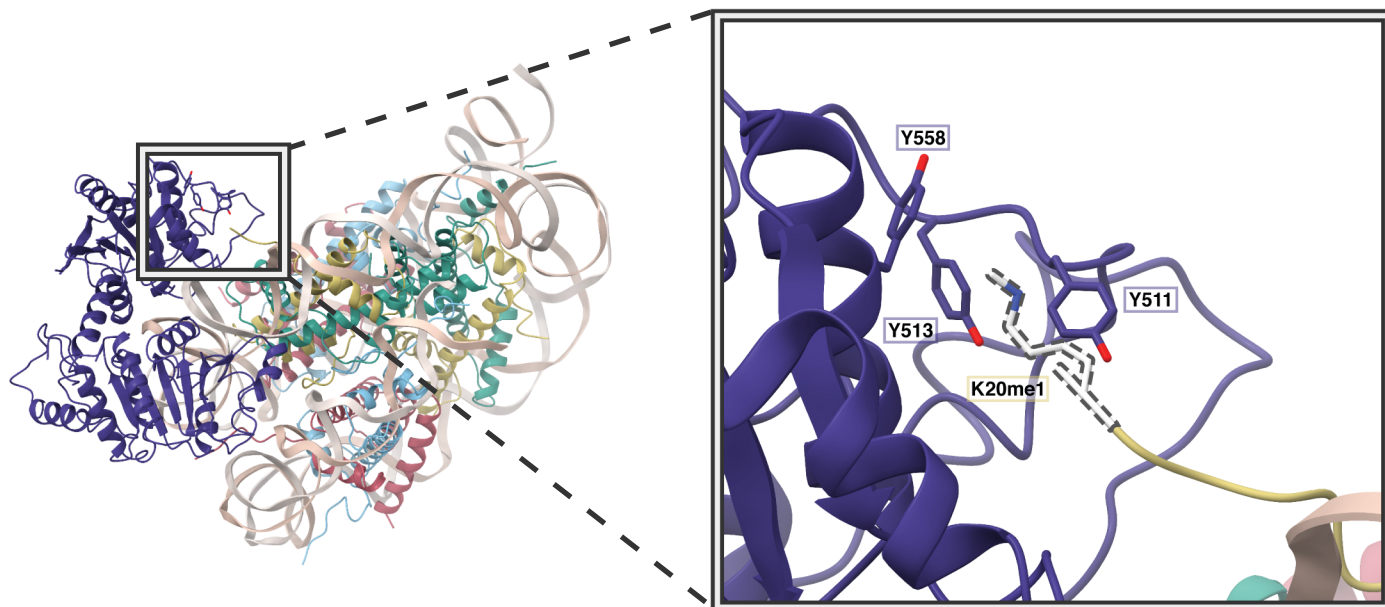

Supplement: 3 — Supplementary Figure S3. Structural comparison of DDM1 with Snf2 family remodelers, related to Figure 4. The structures of (A) Snf2-bound nucleosomes in the absence of nucleotide (PDB code 5X0Y) and (B) Snf2h in the presence of ADP (PDB code 6NE3) (red) superimposed on the structure of DDM1 bound to nucleosome. The Q629R mutation in DDM1 is shown with a red arrow. Alignment was performed using only the nucleosome core particle for each structure. In the presence of bound ADP, the two domains appear in a more closed conformation than the nucleotide free state. The DDM1/nucleosome complex that was reconstructed represents the nucleotide free state. Note that the sample used for the Snf2h structure was prepared with ADP-BeF3 but only ADP was observed in the density. (C) Surface representation of the DDM1-bound nucleosome colored according to degree of DDM1 conservation. Conservation scores were calculated using the Consurf server98 among twenty manually-curated and highly related sequences - such as LSH and HELLS - aligned using Clustal Omega99. The D382A mutation in DDM1 is indicated with a yellow arrow. (D) The electrostatic potential of DDM1 (colored surface) displays a positively-charged groove along the DNA (grey cartoon) interface. Electrostatic surface calculations were performed by APBS100 with a solvent ion concentration of 0.15 M at 298 K using the PARSE force field. (E) The tail of histone H4 extends toward DDM1 such that the residue K20 would be within striking distance of three aromatic residues in DDM1 forming an aromatic cage. The inset indicates a modeled mono-methylated lysine residue with a dashed outline. [file NIHMS1922946-supplement-3.pdf]

**A**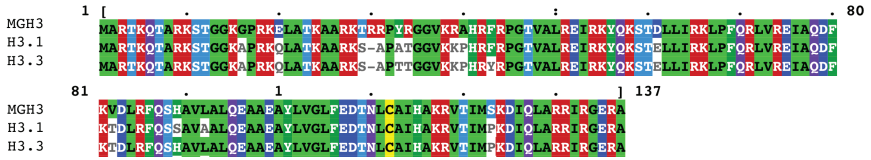**B**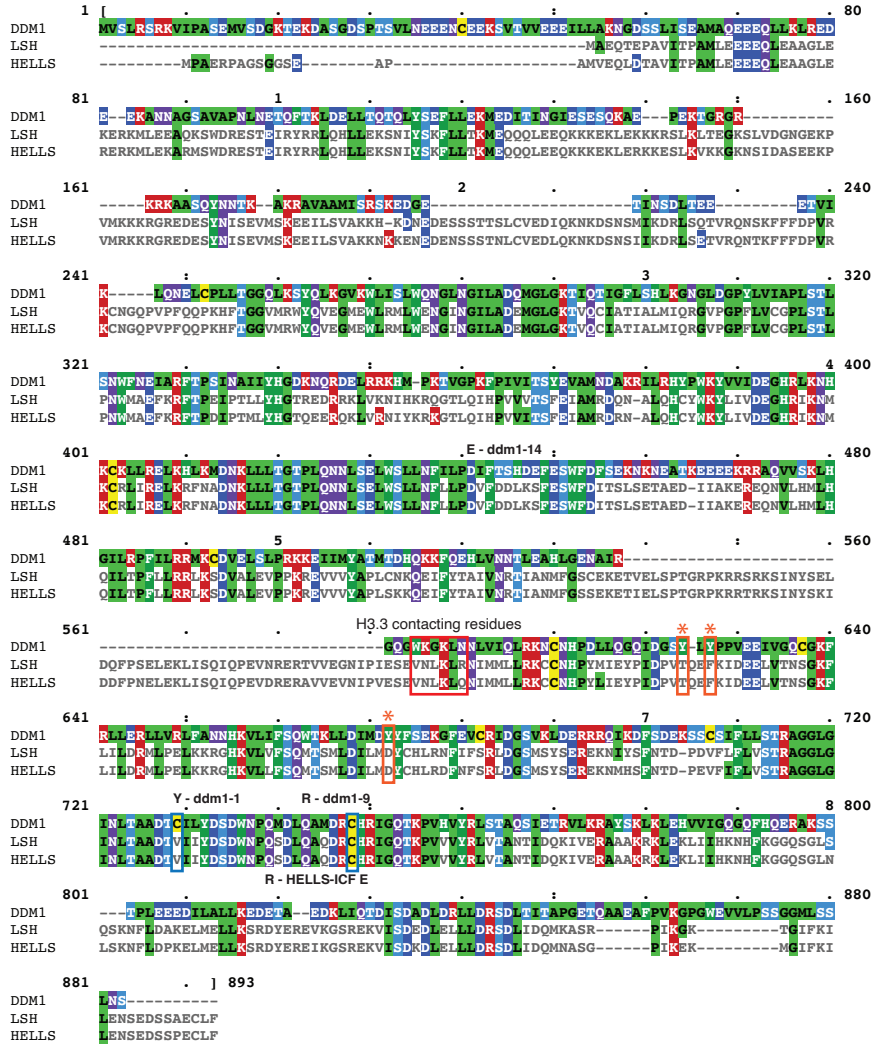**C**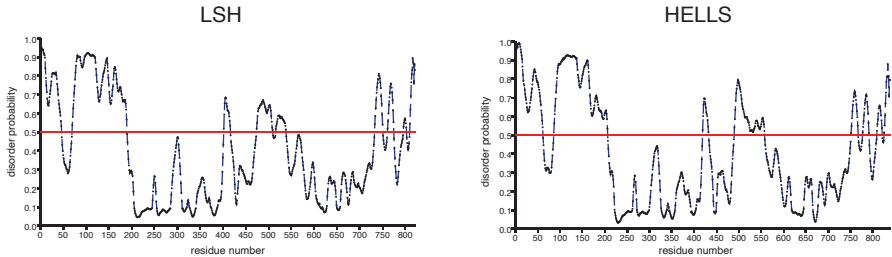

Supplement: 4 — Supplementary Figure S4. Amino acid sequence alignments, related to Figure 4 and 5. (A) The sequence alignment of histone MGH3, H3.1, and H3.3 generated with MView101. (B) The sequence alignment of DDM1, LSH, and HELLS. H3.3 contacting residues (WKGKLN) of DDM1 are indicated with a red bar. Tyrosine residues Y511, Y513, and Y558 (DDM1 aromatic cage) are indicated with orange asterisks. Cysteine residues C615 and C634 (DDM1 disulfide bond) are indicated with blue asterisks. Three DDM1 hypomethylation mutations (ddm1-1, ddm1-9 and ddm1-14) and one HELLS mutation (ICF proband family E) are indicated by substituted residues above and below the mutated location, respectively. Compared to DDM1, LSH has 90.6% coverage with 34.8% identity while HELLS has 92.8% coverage with 33.8% identity. (C) Intrinsically disordered regions of LSH and HELLS using PrDOS102. [file NIHMS1922946-supplement-4.pdf]

**A**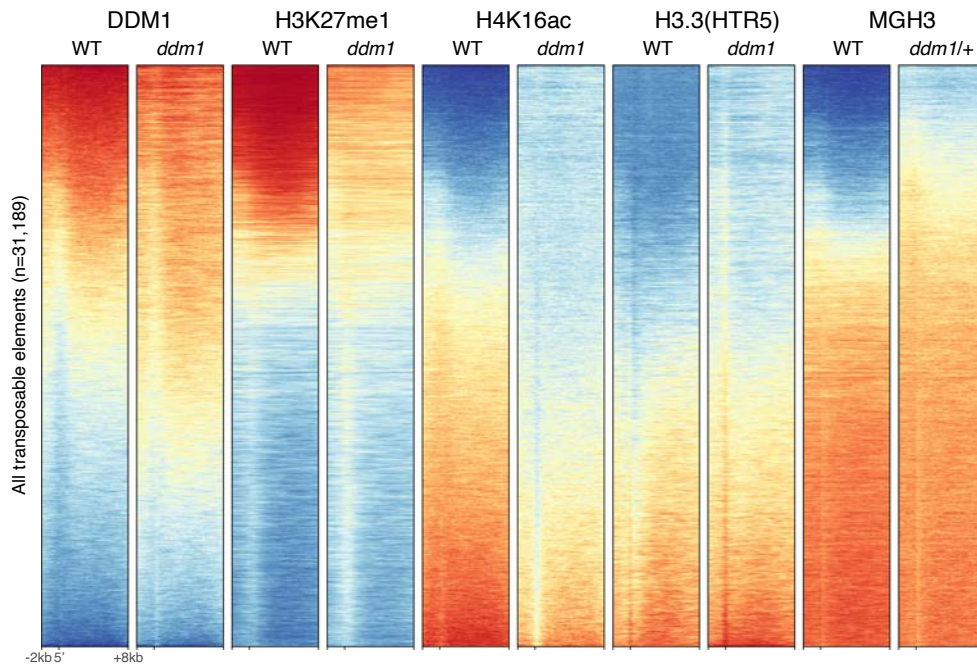**B**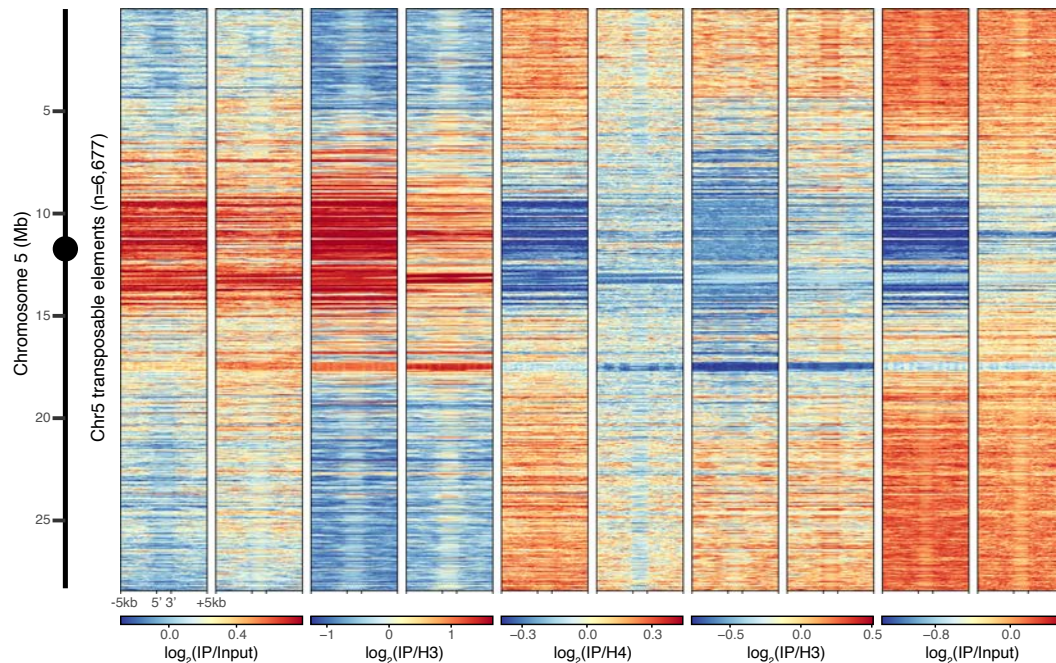

Supplement: 5 — Supplementary Figure S5. ChIP-seq data for all transposable elements in WT and ddm1, related to Figure 6. (A) Heatmaps of DDM1, H3K27me1, H4K16ac, H3.3(HTR5) ChIP-seq of wild-type (WT) and ddm1 genotypes, as well as MGH3 in pollen from WT and ddm1/+ plants, for all transposable elements annotated in TAIR10. Heatmaps were generated using Deeptools95, where all 31,189 TEs were aligned by their 5’ end with 2kb upstream and 8kb downstream with a binsize of 10bp, and sorted based on DDM1 levels in WT. (B) Similar heatmaps were generated using Deeptools, where the 6,677 TEs located on chromosome 5 were scaled to 2kb, represented with 5kb upstream and 5kb downstream with a binsize of 10bp. TEs were kept in order of their location on the chromosome, shown by the scale on the left hand-side. This view highlights that DDM1 preferentially targets peri-centromeric TEs in WT. Both heatmaps highlight correlation between DDM1 and H3K27me1 and anti-correlation with H4K16ac, H3.3 and MGH3 levels in both genotypes, as well as the loss of DDM1 and H3K27me1 from peri-centromeric TEs in ddm1 accompanied by an increase in H4K16ac, H3.3 and MGH3. [file NIHMS1922946-supplement-5.pdf]

**A**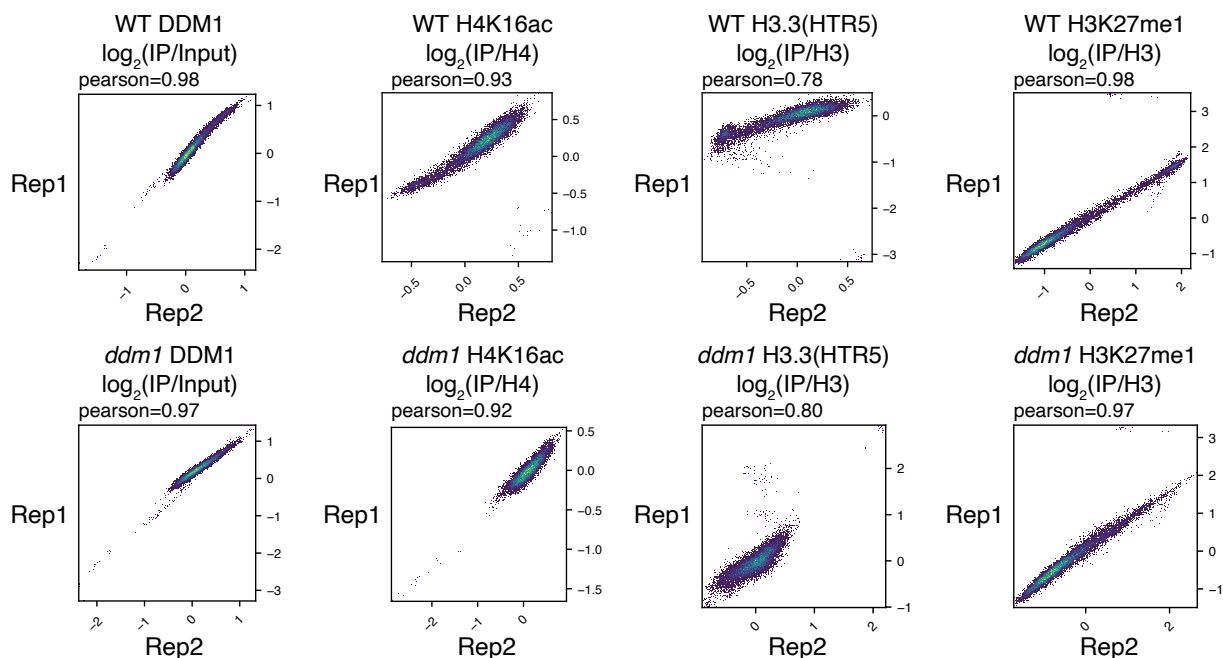**B**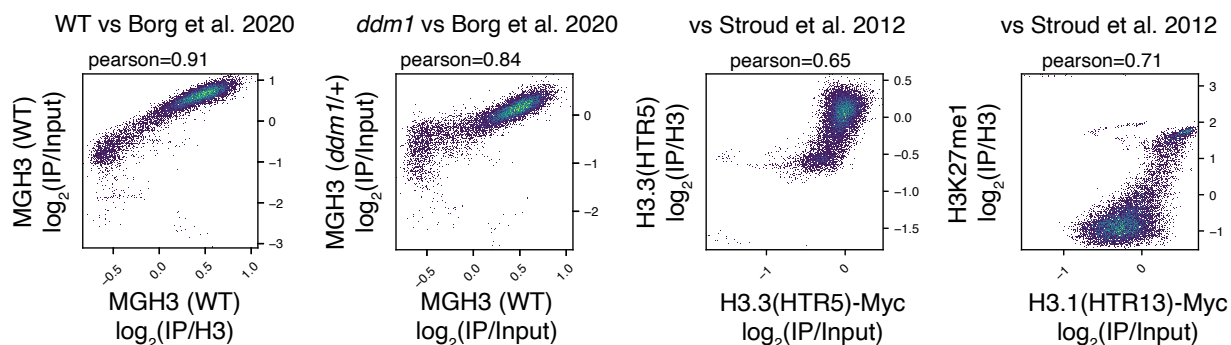**C****D**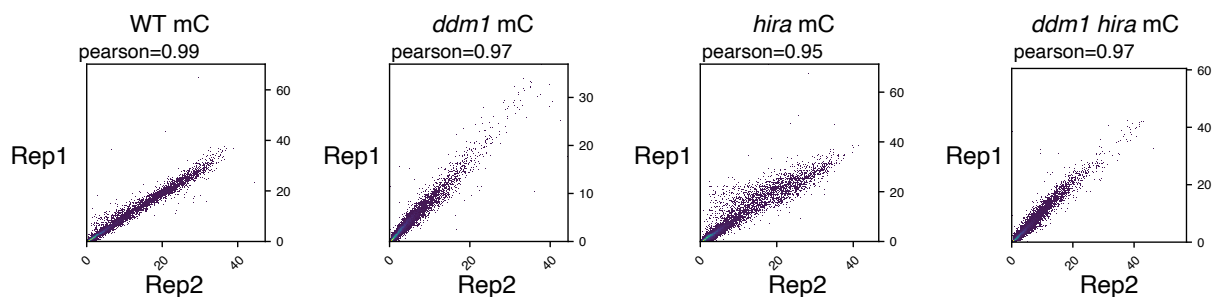**E**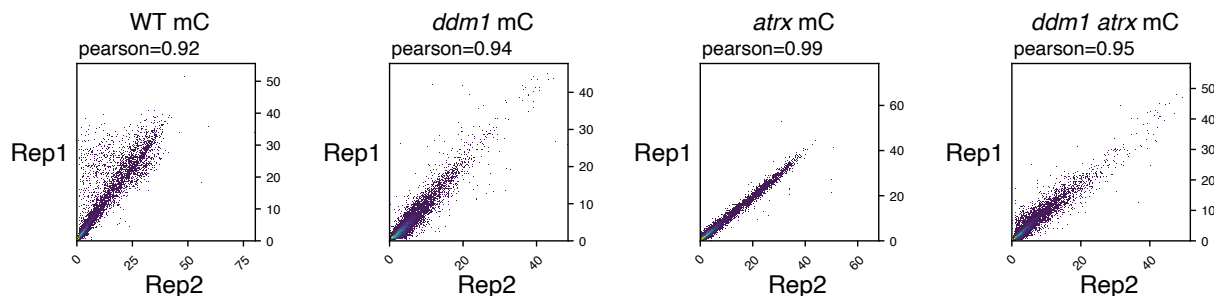

Supplement: 6 — Supplementary Figure S6. Correlations between ChIP-seq replicates and between WGBS replicates, related to STAR Methods. (A) Comparisons of DDM1, H4K16ac, H3.3(HTR5)-RFP and H3K27me1 ChIP-seq data between replicates of each genotype. Pearson correlations are shown. (B) Comparisons of MGH3 in WT and ddm1/+ pollen with previously published MGH3 ChIP-seq47. (C) Comparisons of H3.3(HTR5)-RFP and H3K27me1 ChIP-seq in WT with previously published H3.3(HTR5)-Myc and H3.1(HTR13)-Myc, respectively40. (A-C) Each replicated IP has been normalized to its respective input. (D, E) Comparisons of DNA methylation levels in each replicate for all genotypes grown and processed at the same time for group A (D) and group B (E), respectively. [file NIHMS1922946-supplement-6.pdf]
