# Supplementary material for: Chromatin remodeling of histone H3 variants by DDM1 underlies epigenetic inheritance of DNA methylation
Source: Cell. Author manuscript; Available in PMC 2023 Nov 28. (PMC10529913; doi:10.1016/j.cell.2023.08.001)
Supplement: 8 [file NIHMS1922946-supplement-8.pdf]

Supplementary Table S2. List of mutants in this study, Related to STAR Methods

Mutants	Mution type	Notes
ddm1-2	EMS mutant (nucleotide: G to A)	Hypomorphic; the splicing defect leads to a deletion, a frameshift and premature translation termination; DNA hypomethylation
ddm1-10	T-DNA (SALK_093009)	T-DNA insertion into exon region; used for crossing with MGH3-GFP
met1-1	EMS mutant (amino acid: P 1300 S)	Hypomorphic; amino acid substitution in catalytic domain; DNA hypomethylation; delayed flowering
met1-7	T-DNA (SALK_076522)	T-DNA insertion into exon region; used for crossing with MGH3-GFP
cmt3-11	EMS mutant	Single nucleotide substitution leading to a nonsense mutation; used for crossing with MGH3-GFP
fas2-4	T-DNA (SALK_033228)	Null; no transcript detectable; fasciation
hira-1	WiscDsLox362H05	Reduced fertility
atrx-2	SAIL_861_B04	Reduced fertility

Supplementary Table S3. Primer sequences, Related to STAR Methods

Cloning primers

Name	Primer sequence (5' to 3')	Locus	Note
DDM1_attB1	GGGGACAAGTTTGTACAAAAAAGCAGGCT ATTACTAATTGTGTCGACAAATCC	AT5G66750	for cloning into pDONR221
DDM1_attB2	GGGGACCACTTTGTACAAGAAAGCTGGGT AATCCCAAATCCAAAACATAAGATC	AT5G66750	for cloning into pDONR221
MET1_attB1	GGGGACAAGTTTGTACAAAAAAGCAGGCT TCATGGTAAAATGTTAGTTCTCG	AT5G49160	for cloning into pDONR221
MET1_attB2	GGGGACCACTTTGTACAAGAAAGCTGGGT CTGGACAAACTTTATTTCGAC	AT5G49160	for cloning into pDONR221

Genotyping

Name	Primer sequence (5' to 3')	Locus	Note
ddm1-2 CAPS F	GTTGGACAGTGTGGTAAATTCCGCT	AT5G66750	RsaI digestion
ddm1-2 CAPS R	GAGCTACGAGCCATGGGTTTGTGAAACGT/	AT5G66750	RsaI digestion
ddm1-10 F	GCAAGCCATGGACAGATGCCACAG	AT5G66750	
ddm1-10 R	CAGAGGGCCAATTGTTTTCATCAC	AT5G66750	
met1-1 F	CTCTTTAGTAGAAGTTGGCATG	AT5G49160	HaeIII digestion
met1-1 R	ATATGTATGTATAGATATTTTCTCC	AT5G49160	HaeIII digestion
hira-1 LB	CTACTAAAATTTGAGGCCGGG	AT3G44530	
hira-1 RB	GAGAGTCACTGTTTTGGCTGG	AT3G44530	
atrx-2 LB	AGGAACCCTCACAGCTTCTTC	AT1G08600	
atrx-2 RB	TCACATGGATGGCTTCTTTTC	AT1G08600	
HTR3-GFP-F	TAGTGCAGTCGCAGCTCTTC	AT3G27360	
HTR3-GFP-R	TTTGTACAAGAAAGCTGGGTCAG	AT3G27360	

McrBC-qPCR

Name	Primer sequence (5' to 3')	Locus	Note
ATGP1 F	CGAATGAATCCCTTACCCAAC	AT2G01022	
ATGP1 R	AGCGACATTCGGGAGGAT	AT2G01022	
ATHILA2_1 F	ACCAAGCCGAGTACAACCATAT	AT5G33257	
ATHILA2_1 R	CATTGTGCTCGAGTGTCTGG	AT5G33257	
ATHILA2_2 F	TGCTAGATCGAGTGAGTGTCGT	AT5G35057	
ATHILA2_2 R	CCGAGCCTAGAGAGCAGAAG	AT5G35057	

ChIP-qPCR and RT-qPCR

Name	Primer sequence (5' to 3')	Locus	Note
TSI F	ATCCAGTCCGAAGAACGCGAACTA		
TSI R	TCACTTGTGAGTGTTCGTGAGGTC		
Ta3 F	AAGAGAGCTGGCAGAAGCAGTTGA	AT1G37110	
Ta3 R	ACGCCCTTTACCTTGACCTCCTTT	AT1G37110	
ATHILA6A F	ACAGGAAGTGGGCGCACACC	AT5G32511	
ATHILA6A R	CTCACAACGACGCAAGTGATCT	AT5G32511	
ATHILA2_1 F	ACCAAGCCGAGTACAACCATAT	AT5G33257	
ATHILA2_1 R	CATTGTGCTCGAGTGTCTGG	AT5G33257	
ATHILA2_2 F	TGCTAGATCGAGTGAGTGTCGT	AT5G35057	
ATHILA2_2 R	CCGAGCCTAGAGAGCAGAAG	AT5G35057	

ATPase assay substrates

Name	Primer sequence (5' to 3')	Locus	Note
Widom601 0N60 F	AGAGTGGGAGCTCGGAACACTATCCGAC		
Widom601 0N60 R	CTGGAGAATCCCGGTGCC		

Supplementary Table S4. ChIP and Bisulfite-sequencing libraries metrics, related to STAR Methods.

Genotype	Sample	Group	Replicate	Total reads	All mapped reads	(% total)	Uniquely mapped reads	(% total)	Cytosines covered (WGBS only)	Average coverage (WGBS only)	Non conversion rate (% mC/C in Pt) (WGBS only)
WT	WGBS	A	Rep1	32,230,036	30,942,651	96.01%	24,745,052	76.78%	90.41%	8.17	0.269861
WT	WGBS	A	Rep2	53,613,049	52,389,181	97.72%	42,053,916	78.44%	88.18%	6.78	0.51281
hira	WGBS	A	Rep1	17,362,101	16,566,981	95.42%	12,957,483	74.63%	77.90%	3.93	0.270725
hira	WGBS	A	Rep2	44,991,888	42,569,624	94.62%	33,798,990	75.12%	78.21%	3.22	0.706732
ddm1hira	WGBS	A	Rep1	21,758,809	17,461,284	80.25%	13,837,031	63.59%	77.38%	3.94	0.287453
ddm1hira	WGBS	A	Rep2	48,425,900	36,805,003	76.00%	28,725,572	59.32%	84.11%	6.16	0.548739
ddm1	WGBS	A	Rep1	27,502,435	26,294,284	95.61%	20,404,561	74.19%	83.48%	5.30	0.266461
ddm1	WGBS	A	Rep2	37,878,589	37,470,061	98.92%	29,204,434	77.10%	83.05%	6.49	0.537523
WT	WGBS	B	Rep1	30,561,004	24,364,737	79.72%	18,151,083	59.39%	77.57%	7.07	0.589116
WT	WGBS	B	Rep2	34,550,917	30,417,035	88.04%	22,452,116	64.98%	79.20%	9.83	0.587723
ddm1atrx	WGBS	B	Rep1	23,525,857	19,197,760	81.60%	14,136,528	60.09%	71.88%	6.43	0.595414
ddm1atrx	WGBS	B	Rep2	37,170,647	35,040,723	94.27%	24,906,077	67.00%	79.15%	10.33	0.667431
ddm1	WGBS	B	Rep1	36,520,861	35,544,986	97.33%	26,379,181	72.23%	78.73%	7.54	0.621665
ddm1	WGBS	B	Rep2	20,358,084	19,257,875	94.60%	14,528,944	71.37%	73.49%	4.70	0.923219
atrx	WGBS	B	Rep1	39,661,456	39,246,987	98.95%	29,154,984	73.51%	79.63%	5.21	0.845968
atrx	WGBS	B	Rep2	38,694,008	38,125,949	98.53%	28,077,230	72.56%	79.89%	6.57	0.661121
WT	DDM1	IP	Rep1	40,515,724	37,212,763	91.85%	21,468,053	52.99%	-	-	-
WT	DDM1	Input	Rep1	47,290,771	45,973,121	97.21%	32,105,406	67.89%	-	-	-
WT	DDM1	IP	Rep2	34,645,829	32,569,880	94.01%	19,276,714	55.64%	-	-	-
WT	DDM1	Input	Rep2	51,279,688	49,817,225	97.15%	34,561,985	67.40%	-	-	-
ddm1	DDM1	IP	Rep1	50,102,086	47,183,497	94.17%	27,125,034	54.14%	-	-	-
ddm1	DDM1	Input	Rep1	39,982,483	39,028,473	97.61%	26,984,304	67.49%	-	-	-
ddm1	DDM1	IP	Rep2	49,577,213	43,872,422	88.49%	24,804,809	50.03%	-	-	-
ddm1	DDM1	Input	Rep2	33,380,544	32,612,138	97.70%	22,752,619	68.16%	-	-	-
WT	H3K27me1	IP	Rep1	30,078,663	29,656,796	98.60%	11,522,922	38.31%	-	-	-
WT	H3K27me1	H3	Rep1	22,622,734	22,483,854	99.39%	16,142,362	71.35%	-	-	-
WT	H3K27me1	IP	Rep2	50,994,633	40,503,003	79.43%	13,950,016	27.36%	-	-	-
WT	H3K27me1	H3	Rep2	60,442,954	59,862,967	99.04%	45,680,564	75.58%	-	-	-
ddm1	H3K27me1	IP	Rep1	26,031,998	25,885,357	99.44%	18,435,013	70.81%	-	-	-
ddm1	H3K27me1	H3	Rep1	28,806,975	28,258,079	98.09%	10,926,824	37.93%	-	-	-
ddm1	H3K27me1	IP	Rep2	37,494,713	28,836,811	76.91%	9,554,115	25.48%	-	-	-
ddm1	H3K27me1	H3	Rep2	52,356,409	51,800,730	98.94%	38,115,613	72.80%	-	-	-
WT	H4K16ac	IP	Rep1	29,867,512	29,113,254	97.47%	19,477,351	65.21%	-	-	-
WT	H4K16ac	H4	Rep1	32,025,461	31,151,067	97.27%	17,149,656	53.55%	-	-	-
WT	H4K16ac	IP	Rep2	49,958,674	48,846,091	97.77%	33,687,425	67.43%	-	-	-
WT	H4K16ac	H4	Rep2	49,482,528	48,239,575	97.49%	26,621,503	53.80%	-	-	-
ddm1	H4K16ac	IP	Rep1	44,578,514	43,566,178	97.73%	28,553,612	64.05%	-	-	-
ddm1	H4K16ac	H4	Rep1	59,698,668	20,431,979	34.23%	12,114,825	20.29%	-	-	-
ddm1	H4K16ac	IP	Rep2	40,697,481	39,804,154	97.81%	26,494,041	65.10%	-	-	-
ddm1	H4K16ac	H4	Rep2	33,698,770	32,821,242	97.40%	17,607,160	52.25%	-	-	-
WT	HTR5	IP	Rep1	89,816,986	89,262,349	99.38%	74,274,600	82.70%	-	-	-
WT	HTR5	H3	Rep1	43,362,049	42,608,947	98.26%	33,866,720	78.10%	-	-	-
WT	HTR5	IP	Rep2	47,569,522	47,259,571	99.35%	40,618,380	85.39%	-	-	-
WT	HTR5	H3	Rep2	62,187,338	61,578,996	99.02%	46,846,643	75.33%	-	-	-
ddm1	HTR5	IP	Rep1	91,478,171	90,971,481	99.45%	68,533,360	74.92%	-	-	-
ddm1	HTR5	H3	Rep1	43,901,988	43,551,892	99.20%	31,424,017	71.58%	-	-	-
ddm1	HTR5	IP	Rep2	88,251,305	87,728,045	99.41%	67,051,725	75.98%	-	-	-
ddm1	HTR5	H3	Rep2	77,052,248	76,533,910	99.33%	51,185,737	66.43%	-	-	-
WT	MGH3	IP	Rep1	114,691,469	110,674,811	96.50%	97,134,182	84.69%	-	-	-
WT	MGH3	Input	Rep1	42,239,090	39,899,471	94.46%	25,189,691	59.64%	-	-	-
ddm1 /+	MGH3	IP	Rep1	28,958,163	23,043,589	79.58%	17,882,624	61.75%	-	-	-
ddm1 /+	MGH3	Input	Rep1	25,645,477	24,623,469	96.01%	14,422,150	56.24%	-	-	-

Supplementary Table S5. Cryo-EM data collection and reconstruction statistics for the DDM1-nucleosome complex, Related to STAR Methods

**Data collection**	
Microscope	Titan Krios
Voltage (keV)	300
Magnification	81,000×
Defocus range (μm)	−1.0 to −2.2
Detector	K3
Pixel Size (Å)	1.1
Total exposure ($e^-/Å^2$)	71.2
Exposure rate ($e^-/Å^2$/sec)	14.8
Exposure per frame ($e^-/Å^2$)	2.37
Micrographs collected	8,165

**Initial processing**	
Micrographs used	7,811
Initial particles	3,788,872

**Reconstruction**	
Final particles	215,066
Symmetry	C1
Map sharpening B-factor ($Å^2$)	57.8
Half maps resolution (unmasked / masked)	
FSC 0.143	3.4 / 3.2

Supplementary Table S6. Model refinement and validation statistics for the DDM1-nucleosome complex, Related to STAR Methods

Statistics are provided for the full model as well as the individual octamer, DNA and DDM1 components.

**Refinement**	**Full model**	**Octamer**	**DNA**	**DDM1**
Protein residues	1217	749	——	468
Nucleic acid residues	282	——	282	——
Model resolution (unmasked / masked)				
FSC 0.5	3.2 / 3.2			
FSC 0.143	2.8 / 2.8			
Map correlation coefficients				
CC mask	0.74	0.78	0.73	0.6
CC box	0.65	——	——	——
CC peaks	0.65	——	——	——
CC volume	0.72	0.73	0.72	0.58

**Model geometry**	**Full model**	**Octamer**	**DNA**	**DDM1**
Ramachandran plot				
Outliers (%)	0	0	——	0
Allowed (%)	0.67	0	——	1.72
Favored (%)	99.33	100	——	98.28
Ramachandran plot Z-score				
Whole	−0.32 ± 0.23 (N = 1197)	0.18 ± 0.30 (N = 733)	——	−1.11 ± 0.36 (N = 464)
Helix	0.25 ± 0.19 (N = 681)	0.72 ± 0.23 (N = 492)	——	-0.96 ± 0.34 (N = 189)
Sheet	1.72 ± 0.73 (N = 55)	——	——	1.72 ± 0.73 (N = 55)
Loop	−1.05 ± 0.26 (N = 461)	−1.00 ± 0.36 (N = 241)	——	−1.10 ± 0.38 (N = 220)
CaBLAM outliers (%)	0.4	0.3	——	0.7
Rotamers				
Poor (%)	0.1	0	——	0.24
Favored (%)	98.28	98.72	——	97.62
R.M.S. deviations				
Bond lengths (Å)	0.004	0.004	0.004	0.004
Bond angles (°)	0.759	0.757	0.716	0.837
Geometry outliers				
C_α deviations (%)	0.08	0	——	0.22
C_β deviations	0	0	——	0
Bad bonds	0 / 9,894	0 / 5,991	0 / 6,484	0 / 3,903
Bad angles	0 / 13,296	0 / 8,040	0 / 10,003	0 / 5,256
Cis prolines	0 / 39	0 / 24	——	0 / 15
Chiral volume outliers	0 / 2633	0 / 931	0 / 1,128	0 / 574
Clashscore (all atoms)	3.22	1.97	2.79	5.57
MolProbity score	1.11	0.96	——	1.3
B-factors ($Å^2$) (min / max / mean)				
Protein	12.6 / 98.4 / 45.8	12.6 / 93.9 / 30.7	——	36.1 / 98.4 / 69.2
Nucleotide	28.84 / 201.26 / 87.65	——	28.8 / 201.3 / 87.7	——
